# Cost and cost-effectiveness of four different SARS-CoV-2 active surveillance strategies: evidence from a randomised control trial in Germany

**DOI:** 10.1007/s10198-022-01561-8

**Published:** 2023-01-19

**Authors:** Hoa Thi Nguyen, Claudia M. Denkinger, Stephan Brenner, Lisa Koeppel, Lucia Brugnara, Robin Burk, Michael Knop, Till Bärnighausen, Andreas Deckert, Manuela De Allegri

**Affiliations:** 1 Heidelberg Institute of Global Health, University Hospital and Medical Faculty, Heidelberg University, Im Neuenheimer Feld 130.3, 69120 Heidelberg, Germany; 2 Division of Infectious Disease and Tropical Medicine, Heidelberg University Hospital, Im Neuenheimer Feld 324, 69120 Heidelberg, Germany; 3 German Center for Infection Research (DZIF), Im Neuenheimer Feld 344, Heidelberg, Germany; 4 evaplan GmbH at the University Hospital Heidelberg, Ringstr.19B, 69115 Heidelberg, Germany; 5 Center for Molecular Biology (ZMBH), Heidelberg University, Im Neuenheimer Feld 282, 69120 Heidelberg, Germany; 6 German Cancer Research Center (DKFZ), ZMBH Alliance, 69120 Heidelberg, Germany; 7 Heidelberg Institute of Global Health, University Hospital and Medical Faculty, Heidelberg University, Im Neuenheimer Feld 324, 69120 Heidelberg, Germany

**Keywords:** COVID-19, Surveillance, Cost, Cost-effectiveness, Germany, I180

## Abstract

**Introduction:**

The COVID-19 pandemic has entered its third year and continues to affect most countries worldwide. Active surveillance, i.e. testing individuals irrespective of symptoms, presents a promising strategy to accurately measure the prevalence of SARS-CoV-2. We aimed to identify the most cost-effective active surveillance strategy for COVID-19 among the four strategies tested in a randomised control trial between 18th November 2020 and 23rd December 2020 in Germany. The four strategies included: (A1) direct testing of individuals; (A2) direct testing of households; (B1) testing conditioned on upstream COVID-19 symptom pre-screening of individuals; and (B2) testing conditioned on upstream COVID-19 symptom pre-screening of households.

**Methods:**

We adopted a health system perspective and followed an activity-based approach to costing. Resource consumption data were collected prospectively from a digital individual database, daily time records, key informant interviews and direct observations. Our cost-effectiveness analysis compared each strategy with the status quo and calculated the average cost-effective ratios (ACERs) for one primary outcome (sample tested) and three secondary outcomes (responder recruited, case detected and asymptomatic case detected).

**Results:**

Our results showed that A2, with cost per sample tested at 52,89 EURO, had the lowest ACER for the primary outcome, closely followed by A1 (63,33 EURO). This estimate was much higher for both B1 (243,84 EURO) and B2 (181,06 EURO).

**Conclusion:**

A2 (direct testing at household level) proved to be the most cost-effective of the four evaluated strategies and should be considered as an option to strengthen the routine surveillance system in Germany and similar settings.

**Supplementary Information:**

The online version contains supplementary material available at 10.1007/s10198-022-01561-8.

## Introduction

The global COVID-19 pandemic caused by SARS-CoV-2 has entered its third year and is still affecting most countries. At the time of writing this publication between November 2021 and March 2022, new variants have been detected and hundreds of thousands of new cases are being notified daily [1, 2]. Many countries, including Germany, are experiencing their fifth wave, which has much higher incidence rates than previously reported [2]. Despite steady increases in vaccination coverage globally [3], the uneven distribution of vaccines and the continuous evolution of new variants make the goal of ending the pandemic an extremely difficult and long-term task [1, 4]. Community transmission triggered by a large portion of asymptomatic and pre-symptomatic SARS-CoV-2 carriers (estimated at 42.8%) presents a major challenge for the containment of this highly infectious disease [5–7].

Testing for the presence of SARS-CoV-2 has been identified as a central measure to control the COVID-19 pandemic [8]. Currently, testing is performed for three main purposes: diagnostics, screening and surveillance [9]. Diagnostic testing focuses on accurately identifying individual patients who are infected with SARS-CoV-2 to inform their clinical treatment. Screening testing aims to identify infected individuals in a population, who are then isolated to prevent onward transmission [10]. Meanwhile, surveillance testing serves as a tool to either understand historical exposures (i.e. identifying those previously infected) or to measure ongoing community transmission (i.e. monitoring real-time SARS-CoV-2 spread in communities). Surveillance testing in the form of active surveillance or comprehensive routine surveillance involves testing everyone regardless of their COVID-19 symptoms on a representative sample of a defined population [11]. This form of surveillance enables not only the timely detection of infected individuals, but also improves the monitoring of the disease spread in the community by identifying asymptomatic and pre-symptomatic SARS-CoV-2 carriers. Therefore, it facilitates a more accurate estimate of the true prevalence to better inform the application of preventive and control measures [11, 12]. Given the benefits this strategy carries, the World Health Organization has encouraged countries to implement active surveillance when possible [13].

However, there are few efforts to date to implement active surveillance of SARS-CoV-2 in the general population. We identified only two active surveillance attempts, both of which were conducted in the United Kingdom and made use of real-time reverse transcription-polymerase chain reaction assays (RT-PCR) with swabs self-collected by participants [14, 15]. By providing important estimates on community prevalence of SARS-CoV-2 in different time periods, these two attempts have proved the importance as well as the feasibility of conducting active surveillance for SARS-CoV-2 in high-income settings.

RT-PCR is the current gold standard test for SARS-CoV-2 detection [16]. However, RT-PCR is expensive and often has a turnaround time of 24 to 48 h [17]. In addition, laboratory capacity for RT-PCR testing is often limited, especially in the course of a wave of infection [10]. As a response, novel and rapid SARS-CoV-2 diagnostics, including the reverse transcription loop-mediated isothermal amplification (RT-LAMP) and antigen-based rapid diagnostic tests (Ag-RDTs), along with different self-sampling methods (e.g. saliva, gargle liquid, nasal swabs) have been developed with much lower costs and shorter turnaround times, allowing for expanded testing at the population level [17–21].

Given the general constraints in testing capacity for RT-PCR and the logistic complexity required for population level testing [10], it remains unknown which active surveillance strategy represents a more efficient use of resources. We identified only one modelling study in the United States of America, which showed that expanded surveillance testing (frequent testing of both asymptomatic and symptomatic individuals) using Ag-RDTs is likely to be more cost-effective than the status-quo strategy of testing only symptomatic individuals [22]. In parallel, we found no evidence on the economic costs of implementing surveillance testing of SARS-CoV-2, despite the central role that cost information plays in informing the planning of public health policy and resource allocation [23].

Germany, like most countries, relies mostly on passive surveillance strategies to monitor the spread of SARS-CoV-2 in the general population. This passive form of surveillance involves testing those individuals presenting with clinical COVID-19 symptoms and tracing contact persons in case of a positive test result. Against this background, a team of researchers from Heidelberg University developed an innovative concept for the active surveillance of SARS-CoV-2 in the general population, based on RT-LAMP and self-collected gargle sample. The team tested this concept by conducting a randomized, two-factorial, multi-arm parallel trial (hereafter referred to as Cov-Surv-Study) in the Southwest of Germany [24]. In the trial, four different active surveillance strategies for SARS-CoV-2 were concurrently assessed. The overall aim of the trial was to assess the effectiveness, cost-effectiveness, practicability and acceptability of the four surveillance strategies [24].

As a component of the Cov-Surv-Study trial, our study aimed to strengthen the evidence base on COVID-19 surveillance by assessing the economic costs and cost-effectiveness of the four active surveillance strategies for SARS-CoV-2. Specifically, we estimated the economic cost of establishing and implementing the four active surveillance strategies, using the experimental data collected within the Cov-Surv-Study trial framework. We then assessed the cost-effectiveness of each of the four active surveillance strategies compared with the status quo, i.e. passive or symptom-based surveillance only. By providing reliable estimates on both economic costs and the cost-effectiveness of the four concerned surveillance strategies, our findings are key in shaping further public health policy-making in Germany and in similar settings.

## Methods and data

### Study setting and study intervention

We conducted a costing study and an economic evaluation alongside the Cov-Surv-Study trial. Details of the trial are described elsewhere [24]. In brief, between 18th November 2020 and 23rd December 2020, the trial recruited participants from 56 municipalities in a catchment area with approximately 700,000 inhabitants, covering the city of Heidelberg and the neighbouring Rhine-Neckar district in Germany.

**Fig. 1:**
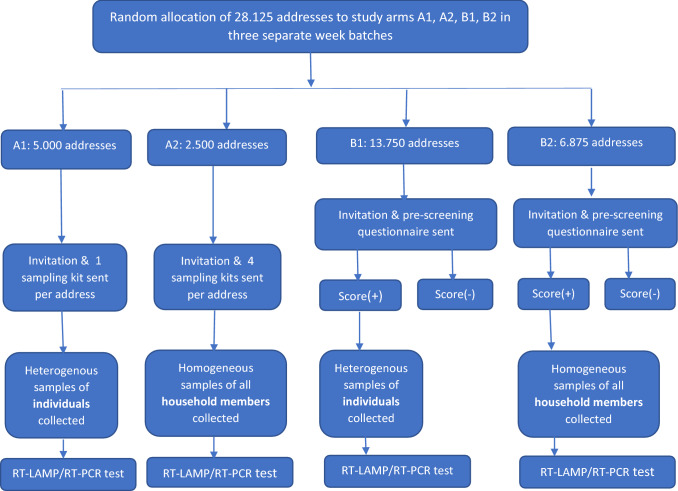
Flow chart of four active surveillance strategies for SARS-CoV-2 tested in the Cov-Surv-Study trial 2020

The Cov-Surv-Study trial evaluated, in its four parallel study arms, four different testing strategies. Specifically, in arm A1, individuals who were randomly selected received an invitation letter per post, which contained the study information, a sampling kit, a voluntary pre-screening questionnaire, and a stamped return envelope. Those who agreed to participate were asked to take a gargle sample by themselves at home after gargling with 5 ml saline solution and to send it back to the indicated laboratory, where RT-LAMP was performed. In arm A2, randomly selected individuals received all the materials as in arm A1, but in sufficient amount (by default four sampling kits, more upon request in the hotline, if needed) to sample all household members. In contrast to arms A1 and A2, individuals in arm B1 first received an invitation letter that contained a pre-screening questionnaire asking about COVID-19 related symptoms and a stamped return envelope. The participants could complete this pre-screening questionnaire online or in paper form. After the questionnaire was analysed, the individuals with positive scores, indicating an increased probability of infection, received a second consignment that contained a test kit. The pre-screening questionnaire (see Annex 1) was developed based on complex machine-learning algorithms as they have shown greater accuracy and efficiency than regression-based methods for the prediction of several diseases [25–28]. This machine learning algorithm, which was used to develop the pre-screening questionnaire, had been trained on datasets containing COVID-19 patients and used to classify participants into COVID-19-free and potentially sick individuals, based on pattern of 16 typical symptoms. The rationale behind the pre-screening questionnaire was to direct testing resources towards those most in need, while covering a wider population. The approach was similar in arm B2, the only difference being that the individual who was contacted first and scored positive in the pre-screening questionnaire subsequently received enough test kits for all household members (again four sampling kits; more upon request in the hotline, if needed). In all arms, all gargle samples that resulted positive to SARS-CoV-2 with RT-LAMP were subsequently confirmed with RT-PCR in a validated diagnostic laboratory of Heidelberg University Hospital. The total sample size of the trial included 28.125 individual contacts and was determined to detect a prevalence of 0,5% at the power of 95% [24]. Figure 1 presents the key contents of the main trial and the four study arms assessed in this analysis.

In addition to setting up mailing logistics for sample collection and laboratory work, four other activities were key for conducting the trial: setting up the study website; development and implementation of an automated IT solution package including a digital individual-level database that tracked every logistic event at the individual level (e.g. invitation sent, test kits sent, pre-screening questionnaire returned, samples returned, tests performed); development and application of the pre-screening questionnaire and an app to screen and score the questionnaire; and setting up and running a hotline to assist the study participants.

### Study perspective and time horizon

We adopted a health system perspective and traced costs from the inception of the trial on 15 September 2020 until its completion on 30 December 2020. Accordingly, we considered all costs borne by the trial implementation team and the local health authorities and categorised costs in two periods: start-up costs incurred from September 15th to November 17th, 2020, and implementation costs incurred from November 18th to December 30th, 2020. All costs were assessed in EURO and in relation to the base year 2020, the time period when the trial was conducted. The cost-effectiveness analysis was informed by the estimates of the costing study and the effects evaluated in the trial. Though the trial included a research component, we excluded all costs related to research activities in our analysis, since these costs are not relevant once a given strategy is adopted for routine implementation. We describe the costing study and the cost-effectiveness analysis in detail below.

### Costing study

We adopted an activity-based micro-costing approach using prospectively collected data [23]. The costing followed three consecutive steps: (i) identification of activities and cost items (resources used), (ii) measurement of resource consumption, and (iii) valuation of costs. We estimated costs for each activity and disaggregated costs by four study arms.

First, in reference to the trial protocol and in close consultation with the trial implementation team, we identified eight main activities for the start-up period and ten activities for the implementation period. Accordingly, the main activities during the start-up phase included: (1) general management; (2) setting up the mailing logistic system (e.g. preparing the protocol for packing study materials, recruitment of personnel, and identifying and selecting the postal service provider); (3) setting up the hotline (e.g. securing rooms, furniture, equipment, and recruitment of personnel); (4) development of trial materials (trial protocol, study materials, invitations etc.); (5) trial design and randomization, (6) development of the pre-screening questionnaire and the screening app; (7) development and establishment of the study website and other IT services, (8) development and setting up IT solutions (the algorithm to record the information on logistic events of study participants automatically in the databases) and the databases of the trial. The implementation period included the same eight activities, which continued after the start-up period. Specifically, the activities during the implementation phase included (1) running the general management; (2) implementing the mailing logistic system, which included printing and packing of study materials by the trial team; (3) running the hotline (e.g. receiving and answering phone calls from study participants; (4) preparing materials for the trial (e.g. press releases, announcement on the local media and power point presentations); (5) sampling and recruitment of study participants; (6) reading and evaluating the pre-screening questionnaires with the screening app; (7) maintaining the study website, (8) monitoring and maintaining the trial databases. Moreover, the implementation phase included two additional activities: sending study materials to the study participants by the contracted postal service provider (postal service for mailings) and the laboratory work, which included packing RT-LAMP test kits (for gargle sample collection), performing RT-LAMP laboratory tests, and performing RT-PCR confirmation tests [18] and (Lou et al. under review).

Second, we measured resource consumption for each activity using multiple tools. For general activities that were carried out at the project overall level (e.g. general management, setting up the mailing logistics, trial design and randomization, etc.), we relied on the daily time records filled by project staff and direct observations in all project meetings by the researchers. Since most staff involved in the trial were employees of Heidelberg University, whose overhead costs for office space and equipment were provided by their respective institutions within the university, we applied an overhead rate of 22% on their time costs, reflecting the standard overhead rate of German public academic institutions [29]. Given the complex set-up of the trial, we conducted several interviews with key project employees to verify information on resource consumption. In addition, we obtained all relevant project documents and financial records to triangulate with the information collected from an individual-level database described above. For activities that were conducted at the individual level (e.g. sending the invitations and test kits, conducting RT-LAMP or RT-PCR tests), we calculated the resource consumption using the individual-level data from the aforementioned digital database of the trial.

We allocated the resources consumed by each arm according to their respective activities. For general activities, which were cross-cutting across all trial arms, making it impossible to trace the use of resources specifically at arm level, we allocated resources evenly across the four arms when we assumed even resource consumption/level of effort and according to level of effort when this was not the case. Particularly, for the start-up cost of the IT solutions and databases, since the IT platform for arms B1 and B2 was designed to accommodate the pre-screening questionnaire and thus was more complex than the one applied to arms A1 and A2 and took 60% of the total staff time, we allocated 60% of the cost of this activity to arms B1 and B2 and the remaining 40% to arms A1 and A2. We assumed that the consumption of IT costs was even between A1 and A2 as well as between B1 and B2. Accordingly, we allocated 20% of IT costs to each of the arms A1 and A2 and 30% to each of the arms B1 and B2. For activities that were conducted at individual-level (test kits sent, tests performed etc.), the allocation relied on the actual use recorded in the individual-level database.

Third, we valued costs by multiplying the resources consumed with the unit prices of these resources. To make our cost estimates proximate to the intended implementation settings, we obtained staff unit cost information from the public salary scale [30]. The cost per hour for each staff member was calculated using the yearly total cost of the equivalent position, including gross salary, costs to the employer. The yearly total cost was then divided by 12 to estimate monthly costs. The monthly cost was further divided by the number of official working days (18,5 days per month, considering national holidays and annual leave), and then by the standard working day of 8 h to obtain the unit cost per hour. For senior personnel advising the overall implementation of the trial, we applied the market consultancy rate for similar seniority level. Information on unit prices of materials was obtained primarily from related invoices.

Since we found no existing information on costs of RT-LAMP test, we conducted our own bottom-up micro-costing study of RT-LAMP, which included both recurrent costs (e.g. staff, reagents, protective gears, and overheads) and capital costs (e.g. building and equipment). We estimated the average cost of RT-LAMP during the trial period to be approximately 8.91 EURO per test, not including the cost for the self-collected gargle sample test kit, which was estimated at 1.05 EURO per kit. We applied these unit costs to estimate the respective cost of RT-LAMP tests performed and the costs of test kits sent. For items that were provided to the trial for free (such as rooms for packing letters and test kits or room for hotline), we used equivalent market prices.

### Cost-effectiveness analysis

#### Base-case analysis using effectiveness estimates of the trial

Our base-case cost-effectiveness analysis related the cost estimates obtained from the costing study described above to the effectiveness measures evaluated in the Cov-Surv-Study trial. Given the central aim of surveillance is to monitor the disease spread in the general population and measure the prevalence, we selected the number of samples tested (one of the four outcome measures of the trial) as the primary outcome measure for our cost-effectiveness analysis. The three remaining outcomes of trial, including the number of responders recruited, the number of cases (both asymptomatic and symptomatic) detected, and the number of asymptomatic cases detected, were adopted as secondary outcomes of our cost-effectiveness analysis. Responders were defined as those who were initially contacted and provided a gargle sample (Arm A1 and A2); or who were initially contacted and answered the pre-screening questionnaire (Arms B1 and B2). The three remaining outcomes (samples tested, cases detected and asymptomatic case detected) were defined identically across arms (Deckert et al., submitted).

To identify the most cost-effective strategy, and given the reality that there is no active surveillance in place in the study setting, we compared all four strategies with the status quo of having no active surveillance. Accordingly, we calculated average cost-effectiveness ratios (ACERs) for the primary outcome as well as for each of the three secondary outcomes, resulting in an average cost per sample tested (primary outcome), an average cost per responder recruited, an average cost per case detected, and an average cost per asymptomatic case detected (secondary outcomes). ACERs for each outcome were calculated by dividing the total costs estimated for each arm by the counts of the corresponding outcomes.

Given that the four active surveillance strategies tested in the trial were implemented in parallel with the existing passive surveillance system managed by the local health authority, it was expected that the individuals who had already been detected as SARS-CoV-2 positive by the passive surveillance would decide not to participate in the trial. During the trial, we actually recorded seven participants (one in A1, three in B1, and three in B2) who called the hotline and declined their invitation to participate in the trial, stating that they had already positively tested within 14 days prior to receiving the invitation. Given that the primary epidemiological outcome of the trial is the 4-week cumulative SARS-CoV-2 prevalence (Deckert et al. under review) and the fact that mild and asymptomatic cases can test positive with RT-PCR until 14 days [31], someone who tested positive within 14 days before the start of the trial might still test positive and hence be counted towards the cumulative four-week prevalence estimated by the trial. We explored this uncertainty by additionally calculating the ACER per case detected with the inclusion of cases reported to the hotline.

#### Sensitivity analyses (SAs)

Since variations in implementation period, response rate and prevalence can greatly influence both costs and effects, we conducted one-way sensitivity analyses on these major drivers of cost-effectiveness results. Specifically, we extended the implementation period from one month as observed in the trial to 12 and 60 months to consider the prolonged nature of the Covid-19 pandemic and thus the need to implement active surveillance for a longer period. During the trial, we observed an overall response rate of 36,6% (range from 34,3% in B2 to 41,2%. in A1). We project that in a routine setting, both lower and higher response rates can be expected because there are differences between a trial set-up and real-life implementation; the two study sites (Heidelberg and the Rhine-Neckar district) are more affluent and have a more highly educated population compared with the national average, so a different response rate may be expected in other geographical locations. We investigated the impact of an uncertain response rate by varying this estimate from 20% to 50% in the sensitivity analysis. In addition, as the prevalence rate changes over time, the number of cases that active surveillance can detect would also change accordingly. We accounted for this uncertainty by varying the prevalence between 0,01% as observed at the onset of the pandemic and 4% as recorded in the months subsequent to the trial closing.

**Fig. 2:**
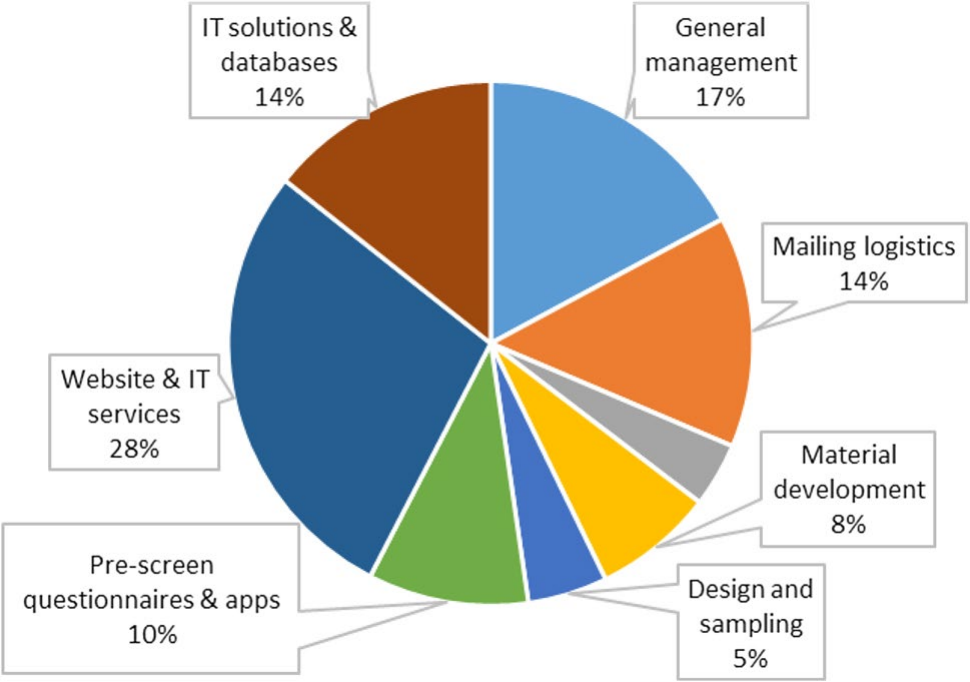
Composition of the start-up costs of the Cov-Surv-Study trial in 2020

Given that all the three major drivers of our cost-effectiveness results examined above can change simultaneously under real-life circumstances and given the fact that other factors (e.g. variations in price of purchased materials or variations in individual-level consumption) can influence costs when the surveillance strategy is implemented in another setting, we built two decision tree models to carry out probabilistic sensitivity analyses (PSAs) on two cost-effective measures, ACER per sample tested and ACER per case detected. Our decision trees have three arms, depicting the strategies A1 and A2 (the two most promising strategies identified from the base-case analyses) with the status-quo A0 of having no active surveillance. The event pathways in each arm followed the logistic flow of the trial and the models were populated using the estimates obtained from the abovementioned costing study and the outcomes measured by the trial. The model structures are described in Figs. 1 and 2 of Annex 2.

Our PSAs relied on a Monte Carlo simulation with 10,000 iterations and considered the joint uncertainty of five influential parameters (i.e. response rate, implementation period, prevalence, start-up costs, and implementation costs) identified from the one-way SAs. In each iteration, model parameters were sampled from the assigned mathematical distributions (gamma for cost parameters and beta or uniform for epidemiological parameters) within the range specified in Table 1 of Annex 2. We present the PSA results as cost-effectiveness acceptability curves that report the probability of an intervention being cost-effective as a function of different willingness-to-pay (WTP) thresholds.

**Table 1:** Economic costs of four SARS-CoV-2 active surveillance strategies in the Cov-Surv-Study trial disaggregated by activity and study phase (start-up and implementation) in 2020

Cost categories	Cost all arms	Quantity of resource used by arm				Cost by arm (EURO)			
	**(EURO)**	**A1**	**A2**	**B1**	**B2**	**A1**	**A2**	**B1**	**B2**
**Start-up costs**									
General management	31.695	25%	25%	25%	25%	7.924	7.924	7.924	7.924
Mailing logistics	26.318	25%	25%	25%	25%	6.580	6.580	6.580	6.580
Hotline	7.316	25%	25%	25%	25%	1.829	1.829	1.829	1.829
Trial material development	13.733	25%	25%	25%	25%	3.433	3.433	3.433	3.433
Design & randomization	9.061	25%	25%	25%	25%	2.265	2.265	2.265	2.265
**Pre-screening questionnaire & apps**	**18.280**	**n/a**	**n/a**	**50%**	**50%**	**n/a**	**n/a**	**9.140**	**9.140**
Website & IT services	51.858	25%	25%	25%	25%	12.965	12.965	12.965	12.965
**IT solutions & databases**	**26.472**	**20%**	**20%**	**30%**	**30%**	**5.294**	**5.294**	**7.942**	**7.942**
**Subtotal start-up costs**	**184.734**					**40.290**	**40.290**	**52.077**	**52.077**
**Implementation costs**									
General management	24.138	25%	25%	25%	25%	6.034	6.034	6.034	6.034
**Mailing logistics**	**96.091**	**4.962**	**2.481**	**13.644**	**6.821**	**30.642**	**21.086**	**26.986**	**17.326**
**Postal services**	**79.834**	**12.617**	**6.249**	**30.319**	**15.156**	**20.749**	**10.234**	**32.586**	**16.264**
**Hotline**	**14.866**	**516**	**314**	**840**	**478**	**3.571**	**2.173**	**5.814**	**3.308**
Trial material development	2.324	25%	25%	25%	25%	581	581	581	581
Sampling and recruitment	261	25%	25%	25%	25%	65	65	65	65
Pre-screen questionnaires & apps	5.723	n/a	n/a	50%	50%	n/a	n/a	2.861	2.861
IT solutions & databases	8.321	25%	25%	25%	25%	2.080	2.080	2.080	2.080
RT-LAMP test kits for sample collection	18.151	4.962	9.924	809	1.592	5.210	10.420	849	1.672
RT-LAMP laboratory tests	47.499	2.031	2.148	555	597	18.096	19.139	4.945	5.319
RT-PCR confirmation tests	3.788	26	30	9	10	1.313	1.515	455	505
**Subtotal implementation costs**	**300.995**					**88.343**	**73.328**	**83.256**	**56.016**
**Total costs**	**485.729**					**128.633**	**113.618**	**135.334**	**108.093**

Finally, since the number of cases detected by arms B1 and B2 was dependent on the performance of the pre-screening tool, and since it was not possible to ascertain the sensitivity of our pre-screening questionnaire due to time constraints (less than 2 months for development and testing the pre-screening questionnaire) and lower response rates than originally envisaged in the trial, we ran a separate sensitivity analysis to estimate the number of cases detected for arms B1 and B2 by using the sensitivity of 90% reported for a validated pre-screening questionnaire in Israel [32] and the sensitivity of RT-LAMP at 97.5% [18].

## Results

Following the sequence of our analyses, we first report results of our costing study. Second, we present the cost-effectiveness results of the base-case analysis using the data from the trial. Third, we report results of one-way sensitivity analyses on the four most influential parameters driving our cost-effectiveness estimates: extended implementation period to 12 months and 60 months; varying response rate from 20 to 50%; increasing prevalence from 0,4% to 1% and 4%; and setting the sensitivity of the pre-screening tool at 90%. Last, we report PSA results on cost per sample tested and cost per case detected.

## Economic cost of four SARS-COV-2 active surveillance strategies in the Cov-Surv-Study trial

In Table 1, we present the economic costs of the four SAR-CoV-2 active surveillance strategies tested in the Cov-Surv-Study trial, disaggregated by activity and study phase (start-up and implementation). Overall, the trial incurred a total cost of 485.729 EURO, which included a start-up cost of 184.734 EURO (38% of total cost) and an implementation cost of 300.995 EURO (62% of total cost). Arm B2 accounted for the lowest share of the cost (108.093 EURO or 22% of the total cost), followed by A2 (113.618 EURO, 23%), A1 (128.633 EURO, 26%) and B1 (135.334 EURO, 27%).

**Fig. 3:**
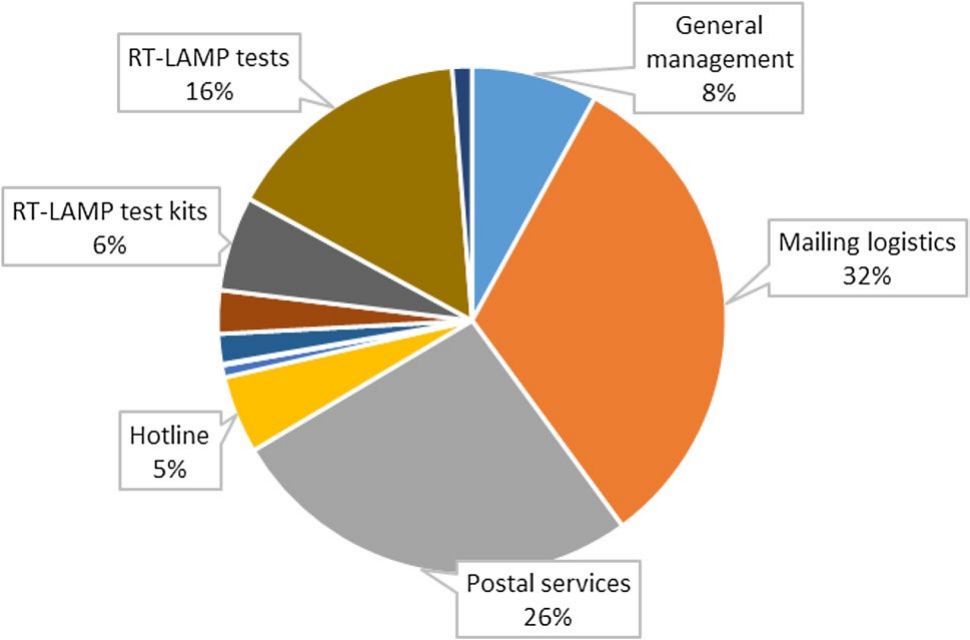
Composition of the implementation costs of the Cov-Surv-Study trial in 2020

We visualize the composition of start-up costs in Fig. 2 and the composition of implementation costs in Fig. 3. For clarity, we do not show the label for activities, which accounted for less than 5% of the total costs. During the start-up period, the website set-up and other IT services accounted for the largest share of the cost (28%), followed by general management costs (17%), IT solutions & databases (14%), mailing logistics (14%) and development of the pre-screening questionnaire and an app to automate the pre-screening questionnaire evaluation (10%). The start-up cost of the remaining three activities (setting up the hotline, trial design and randomization, and development of study materials) accounted for 4%, 5% and 8%, respectively.

During the implementation period, mailing logistics, postal services for mailings and RT-LAMP testing were the three activities accounting for the major share of costs at 32%, 26%, and 16%, respectively. General management, RT-LAMP test kits and the hotline accounted for a much lower share of costs at 8%, 6% and 5%, respectively.

## Cost-effectiveness of the four SARS-CoV-2 surveillance strategies: base-case analysis results

Our cost-effectiveness analyses used the effectiveness estimates measured by the trial. The methods and results of the effectiveness analysis were reported in the main trial paper (Deckert et al., submitted). To facilitate the comprehension of our cost-effectiveness results, in Table 2, we present the effectiveness results together with our base-case cost-effectiveness estimated for the response rate and the prevalence observed for each arm during the trial. We made an exception when it came to the number of asymptomatic cases detected, since we did not use the estimates reported in the trial. The reason for this exception was that answering the pre-screening questionnaire was voluntary in arms A1 and A2, therefore, it was impossible to identify all the asymptomatic cases detected in these two arms. Instead, we derived the number of asymptomatic/pre-symptomatic cases that could have been detected in arms A1 and A2 by applying the middle value (42,5%) of the range estimates (40%-45%) on the proportion of asymptomatic cases reported in a systematic review on the prevalence of asymptomatic/pre-symptomatic cases to the number of all cases detected [33].

**Table 2:** Cost-effectiveness of four SARS-CoV-2 surveillance strategies calculated for the observed response rate and the observed prevalence

Effectiveness results	Surveillance strategies			
	**A1**	**A2**	**B1**	**B2**
Response rate (36,6% overall)	41,2%	36,2%	36,1%	34,4%
Observed cumulative four-week prevalence	0,31%	0,35%	0,07%	0,02%
**Outcome estimates**				
Number of responders recruited	2.043	898	4.926	2340
Number of samples tested***	2.031	2.139	555	594
Number of cases detected	6	7	3	1
Number of cases could have been detected*	1	0	3	5
Number of asymptomatic cases	3	3	n/a	n/a
**Average cost per outcome (EURO)**				
Cost per responder recruited	62,96	126,52	27,47	46,19
**Cost per sample tested**	63,33	53,12	243,84	181,06
**Cost per case detected**	21.439	16.231	45.111	108.093
Cost per case detected all**	18.376	16.231	22.558	27.023
Cost per asymptomatic case detected	42.878	37.873	n/a	n/a

^*^ *Cases which reported to test positive two weeks before the trial and therefore declined to participate*

^**^ Including cases which have declined to participate because of having positive test two weeks before the trial

^***^ Consisting of samples provided by responders (A1 and B1) and their household members (A2 and B2)

With regard to the primary outcome measure (samples tested), A2 has the most favourable ACER with cost per sample tested at 52,89 EURO, closely followed by A1 with cost per sample tested at 63,33 EURO. Both B1 and B2 had much higher cost per sample tested at 243,84 EURO and 181,06 EURO respectively. With regard to the secondary outcomes (responders recruited, cases detected and asymptomatic cases detected), B1 had the lowest cost per responder (27,47 EURO), consecutively followed by B2 (40,03 EURO), A2 (51,64 EURO) and A1 (62,96 EURO). Cost per case detected was lowest for A2 (16.231 EURO), followed by A1 (21.439 EURO), B1 (45.111 EURO) and finally B2 (108.093). Cost per asymptomatic case was lower for A2 (37.873 EURO) than for A1 (42.878 EURO).

When adding the cases that could have been detected by the trial to consider the scenario where there would be no overlap between the existing passive surveillance and the tested active surveillance system as explained in the method session, cost per case detected remained the same for A2 (16.231 EURO) but much lower for A1 (down to 18.376,14 EURO from 21.439 EURO), B1(down to 22.558 EURO from 45.111 EURO) and B2 (down to 27.023 EURO from 108.093 EURO).

## Sensitivity analysis (SA) results

### One-way SA results of extending the implementation periods to 12 months and 60 months

When extending the implementation period from 1 month to 12 months while keeping all other parameters (e.g. response rate, prevalence and resource consumption by arms) constant as of the trial period, the start-up costs remained the same as the base-case estimates. Meanwhile, the implementation costs increased by 12 for almost all activities except for mailing postal costs, which increased by 16. The exception of the postal costs was made to reflect the fact that the invitations and sending of sampling kits were made in three separate weekly batches, while other activities (e.g. laboratory work, general management and hotline) lasted for about a month as described in the method section and reported in the main trial paper (Deckert et al., submitted). As a result, when extending the implementation period to 12 months, the estimated ACERs were lower for all outcomes across all four strategies as shown in Table 3. Still, A2 remained the strategy with the most favourable ACER per sample tested as well as per case detected (37,29 EURO and 11.442 EURO), closely followed by A1 (48,56 EURO and 14.088 EURO). When extending the implementation period to 60 months, the ACER per sample tested and per case detected for A2 reduced to 36,19 EURO and 11.059 EURO respectively. Detailed results of the one-way SA results when extending the implementation period to 60 months were provided in Annex 3.

**Table 3:** Cost-effectiveness results estimated for the implementation period extended to 12 months

Cost-effectiveness results	Surveillance strategies			
	**A1**	**A2**	**B1**	**B2**
**Cost estimates in EURO**				
Start-up costs	40.290	40.290	52.077	52.077
Implementation costs	**1.143.116**	**920.870**	**1.129.421**	**737.254**
Total costs	**1.183.406**	**961.160**	**1.181.498**	**789.331**
**Outcome estimates**				
Number of responders recruited	24.516	10.776	59.112	28.080
Number of samples tested	24.372	25.668	6.660	7.128
Number of cases detected	72	84	36	12
Number of cases could have been detected*	12	0	36	36
Number of asymptomatic cases	36	36	n/a	n/a
**Average cost per outcome in EURO**				
Cost per responder recruited	48,27	89,19	19,99	28,11
Cost per sample tested	48,56	37,45	177,40	110,74
Cost per case detected	**16.436**	**11.442**	**32.819**	**65.778**
Cost per case detected all**	**14.088**	**11.442**	**16.410**	**16.444**
Cost per asymptomatic case detected	32.873	26.699	n/a	n/a

^*^ *Cases which reported to test positive two weeks before the trial and therefore declined to participate*

^**^ Including cases which have declined to participate because of having positive test 2 weeks before the trial

### The one-way SA results of increasing the prevalence to 1% and 4%

When the prevalence varied between 0.01% and 4%, the cost per case detected increased or decreased proportionally. Specifically, when the prevalence reduced to 0.01% from 0.31%, 0.35%, 0.07% and 0.02% for arms A1, A2, B1 and B2 respectively (estimated for each arm during the trial period the trial), the cost per case detected increased dramatically and proportional to the reduction level of the hypothetical prevalence (i.e. 31 times for A1, 35 times for A2, 7% for B1 and 2% for B2). In the contrary, when the prevalence increased to 4% across four arms, the cost per case detected decreased substantially and in proportion with the increase level of the hypothetical prevalence (i.e. 1290 times for A1, 1143 times for A2, 5714 times for B1 and 20.000 times for B2). Table 4 reports detailed results of the one-way SA analysis when the prevalence varied between to 0.01% and 4%.

**Table 4:** Cost-effectiveness results of four SARS-CoV-2 surveillance strategies estimated when the prevalence varied between 0.01% and 4%

Effectiveness results	Surveillance strategies			
	**A1**	**A2**	**B1**	**B2**
Estimated cumulative 4-week prevalence	0,31%	0,35%	0,07%	0,02%
*At prevalence of 0.01%*				
Number of cases detected	0,19	0,20	0,43	0,50
Number of cases could have been detected*	0,03	0,00	0,43	1,50
Number of asymptomatic cases detected	0,1	0,09	n/a	n/a
*At prevalence of 4%*				
Number of cases detected	77,42	80	171,43	200
Number of cases could have been detected*	12,90	0,00	171,43	600
Number of asymptomatic cases detected	38,39	34	n/a	n/a
**Cost-effectiveness results**	**A1**	**A2**	**B1**	**B2**
*At prevalence of 0.01%*				
Cost per case detected	**664.604**	**568.088**	**315.778**	**216.187**
Cost per case detected all**	**569.660**	**568.088**	**157.889**	**54.047**
Cost per asymptomatic case detected	1.329.208	1.325.539	n/a	n/a
*At prevalence of 4%*				
Cost per case detected	**1.662**	**1.420**	**789**	**540**
Cost per case detected all**	**1.424**	**1.420**	**395**	**132**
Cost per asymptomatic case detected	3.351	3.341	n/a	n/a

^*^ *Cases which reported to test positive two weeks before the trial and therefore declined to participate*

^**^ Including cases which have declined to participate because of having positive test two weeks before the trial

### The one-way SA results of varying the response rate from 20 to 50%

When the response rate increased to 50%, the ACERs decreased for all outcomes across all four arms. For example, the cost per sample tested and the cost per case detected for A2 went down to 45,45 EURO and 13.947 EURO respectively. In contrast, when the response rate decreased to 20%, the cost per sample tested increased to 51,53 EURO and the cost per case detected increased to 18.869,03 EURO. The detailed one-way SA results when the response rate increased to 50% are presented in Table 5, and the one-way SA results when the response rate decreased to 20% are presented in Annex 3.

**Table 5:** Cost-effectiveness results of four SARS-CoV-2 surveillance strategies estimated for the response rate increased to 50%

Effectiveness results	Surveillance strategies			
	**A1**	**A2**	**B1**	**B2**
Number of responders recruited	2.223	1.022	5.611	2.705
Number of samples tested	2.210	2.434	632	687
Number of cases detected	7	8	3	1
Number of cases could have been detected*	1	0	3	3
Number of asymptomatic cases detected	3,40	3,40	n/a	n/a
**Average cost per outcome**				
Cost per responder	56,84	108,72	24,02	39,71
Cost per sample tested	57,18	45,64	213,17	156,42
Cost per case detected	**19.355**	**13.947**	**39.436**	**92.911**
Cost per case detected all**	**16.590**	**13.947**	**19.718**	**23.228**
Cost per asymptomatic case detected	37.163	32.677	n/a	n/a

^*^ *Cases which reported to test positive two weeks before the trial and therefore declined to participate*

^**^ Including cases which have declined to participate because of having positive test two weeks before the trial

### PSA results on cost per sample tested and cost per case detected

**Fig. 4:**
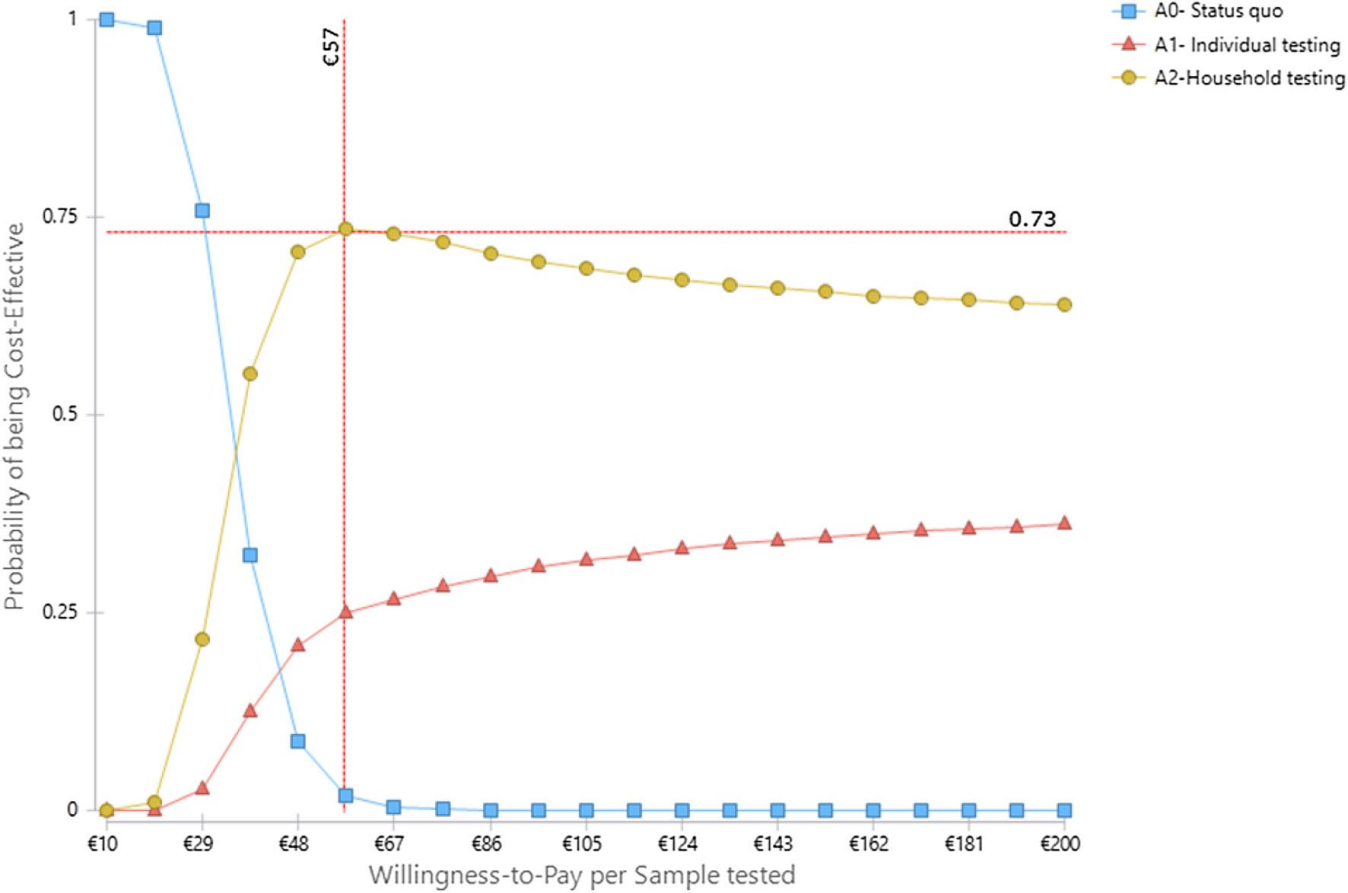
Cost-effectiveness acceptability curves at different willingness-to-pay per sample tested for the strategies A1, A2 and the status quo A0 in the Cov-Surv-Study trial in 2020

Figure 4 visualizes the PSA results on cost per sample tested for the strategies A1, A2 and the status quo of having no active surveillance. Both A1 and A2 are more effective than A0 but at a higher cost. A2 extendedly dominates A1 at all levels of willingness to pay. At a willingness to pay of 57 EURO per sample tested, A2 has a 73% probability of being more cost-effective than both A0 and A1.

**Fig. 5:**
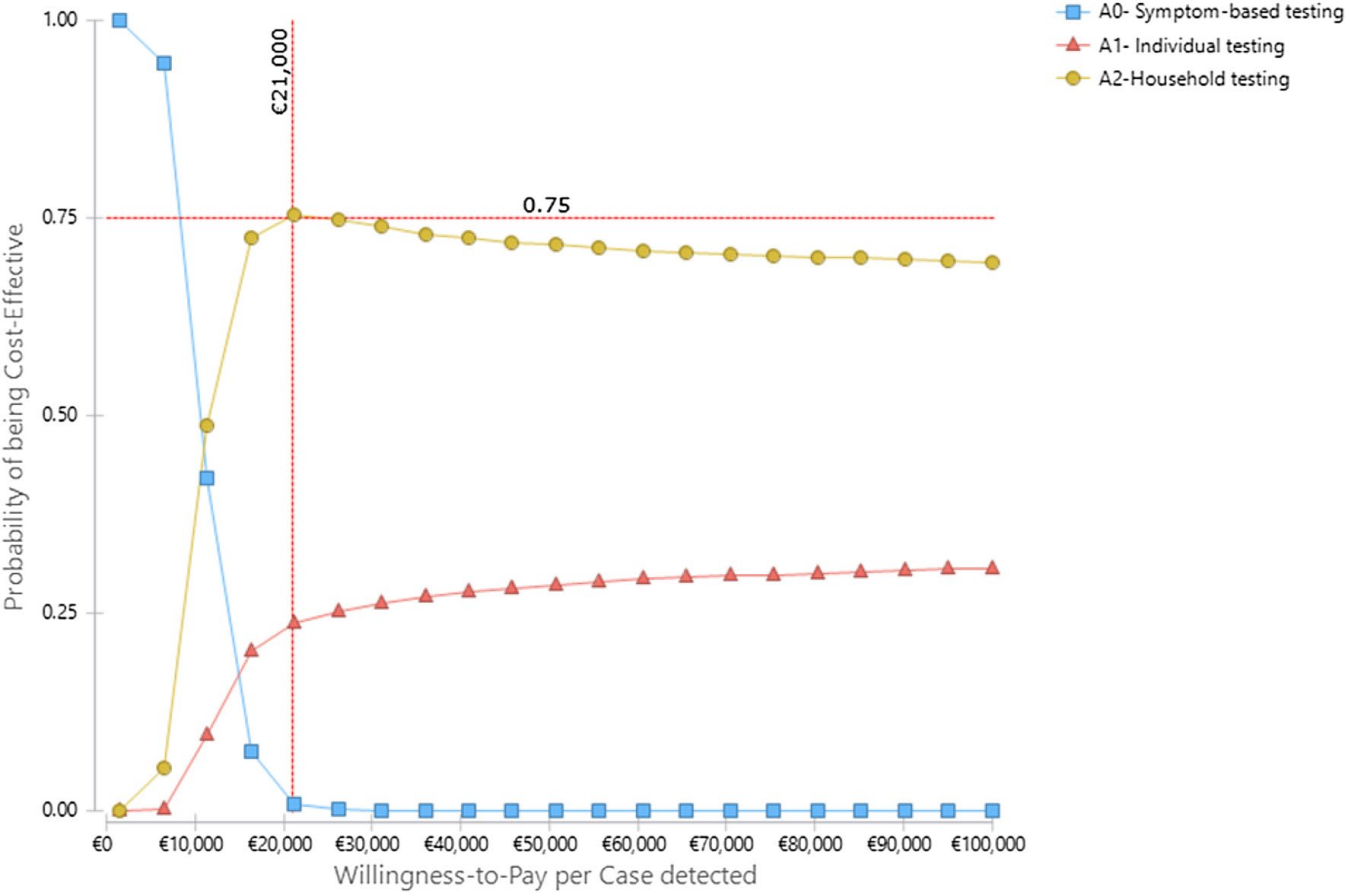
Cost-effectiveness acceptability curves at different willingness-to-pay per case detected for the strategies A1, A2 and the status quo A0 tested in the Cov-Surv-Study trial in 2020

Similarly, Fig. 5 visualizes the PSA results on cost per case detected for the strategies A1, A2 and the status quo A0. Both A1 and A2 are more effective than A0 but at a higher cost. A2 extendedly dominates A1 at all level of willingness to pay. At the willingness to pay of 19.000 EURO per sample tested, A2 has a 73% probability of being more cost-effective than both A0 and A1.

### SA result of applying the sensitivity of 90% to the pre-screening questionnaire

When applying the sensitivity of 90% to the pre-screening questionnaire, and assuming the same prevalence (0,07% for B1 and 0,02% for B2) between responders and non-responders, the cost per case detected was substantially lower for both B1 (a reduction from 22.556 EURO to 11.360 EURO) and B2 (a reduction from 27.023 to 17.334 EURO).

## Discussions

To our knowledge, our study represents the first attempt worldwide to assess the economic costs and cost-effectiveness of four concurrent active surveillance strategies for SARS-COV-2 in a high-income setting. The Cov-Surv-Study trial offered us a unique opportunity to collect high-quality experimental data on both costs and outcomes for our cost-effectiveness analysis. Both our base-case analysis and multiple sensitivity analyses (extending the implementation period, varying response rates, and increasing prevalence) consistently showed that the strategy A2, i.e. direct testing of randomly selected households, is the most cost-effective of the four tested strategies. When comparing strategy A2 with the status quo of having no active surveillance, the average cost per sample tested (the primary outcome of our cost-effectiveness analysis) was estimated at 52,89 EURO. Strategy A1, i.e. directing testing of randomly selected individuals, closely followed strategy A2 with an average cost per sample tested of 63,33 EURO. Both strategies B1, i.e. testing conditional upon upstream pre-screening of symptoms of randomly selected individuals, and B2, i.e. testing conditional upon upstream pre-screening of symptoms of randomly selected households, had much higher ACERs per sample tested than A1 and A2.

Given the novelty of our study, appraising findings against existing literature on the cost-effectiveness of COVID-19 active surveillance is not possible. Still, our findings identifying A2 as the most cost-effective strategy suggest that economies of scale can be achieved when testing entire households instead of single individuals across households. Further, our sensitivity analysis suggests that extending the implementation period to 12 months could result in a reduction in costs of 30%, bringing the cost per person tested as low as 37,29 EURO. This finding is important because it suggests that if strategy A2 were to be adopted for routine implementation as a long-term effort, its cost per sample tested would be substantially lower. Furthermore, our cost computation included a unit cost of 8,91 EURO for each RT-LAMP test performed, which reflects the test cost at the time of the trial, when the laboratory was used at most at 50% capacity. Meanwhile, our cost study of the RT-LAMP test suggests that this unit cost could decrease to 6,29 EURO per test when the laboratory was utilized at 100% capacity. This would make the implementation of the RT-LAMP based surveillance strategy under routine conditions even cheaper because, in routine implementation, it is more likely to make an accurate projection of the expected number of tests and therefore facilitate the optimal use of laboratory capacity.

Given that population surveillance aims to monitor the spread of SARS-CoV-2 and concurrently measure the true magnitude of the disease so as to inform the application of appropriate prevention and control measures [34, 35], the ability to detect asymptomatic cases is a fundamental requirement for an effective population surveillance system. Our analysis estimated that strategy A2 detected an asymptomatic SARS-CoV-2 infection case at an average cost of 16.231 EURO at a prevalence of 0,4%. This estimate could be reduced to 3.819 EURO if the prevalence increased to 4%. Since there is no explicit cost-effectiveness threshold to assess the value of public health interventions in Germany, we appraised the value of detecting asymptomatic SARS-CoV-2 cases in the general population in relation to pandemic control practices in the country. At the time of the trial, the prevalence was very low (estimated at 0,4%) and thus contract tracing efforts by local health authorities could have been very effective and capable of identifying almost all cases, leading to an accurate estimate of the true prevalence. However, the dramatic surge in cases due to Omicron since the beginning of 2022 has made contract tracing almost impossible. In this instance, without active surveillance, the passive surveillance would miss a large portion of asymptomatic and pre-symptomatic SARS-CoV-2 carriers, leading to the underestimation of the prevalence and the onward transmission of the virus in the community.

Our finding that strategies B1 and B2 have lower average cost per responder, but much higher cost per sample tested and case detected than A1 and A2, suggests that the pre-screening questionnaire is likely to have performed less well than expected. To investigate the impact of this uncertainty on the performance of B1 and B2, we conducted a sensitivity analysis using the sensitivity of 90% reported for a validated pre-screening tool in Israel, and found that the cost per case detected decreased substantially in both B1 and B2. However, given the focus of active surveillance on the early detection of cases and detection of asymptomatic cases and the large proportion of asymptomatic cases (40%–45%) of COVID-19 [33], the use of pre-screening tools in active surveillance should be only considered in settings where there is an extreme scarcity of testing capacity and thus the need to allocate available tests to the population most at risk. Furthermore, our one-way SA varying the prevalence from 0,01% to 4% showed a much favourable cost per case detected for both B1 and B2 relative to A1 and A2. This finding points to the fact that the unfavourable cost per case detected for B1 and B2 estimated in the base-case analyses was mainly driven by the much lower prevalence observed for B1 (0,07%) and B2 (0,02%) in comparison to the estimates for A1 (0,31%) and A2 (0,35%) during the trial period.

Of note, our cost composition analysis indicated that start-up costs accounted for 38% of the total costs, with the cost to set up the website and other IT services taking up the largest share (28%). Given the short implementation period of the trial (1 month), these costs appeared substantial and may suggest a cost structure imbalance. In routine implementation, with active surveillance being implemented at scale, infrastructure (website, automated database, screening app etc.) costs would be distributed over a much longer period, resulting in increased affordability.

Our findings should be appraised against several methodological considerations. First, our cost data were obtained within the trial framework in which the logistic preparations were made with the highest possible standards, thus the estimated costs might be higher than in a routine setting. Second, our study was conducted in only two districts, and therefore, our findings have a limited generalizability within and outside Germany. However, to facilitate the use of our cost estimates beyond the study settings, we obtained the unit cost of project staff from the public salary scale, which is applied almost uniformly in Germany, and the actual purchased prices of the mailing materials, which have a negligible variation across Germany. Furthermore, we developed two decision tree models and conducted PSAs on five important factors which can influence the cost and cost-effectiveness when the adopted strategy is implemented in other geographical locations or in routine conditions. Third, the overall response rate observed in the trial was 36,6%, which was much lower than the initially expected 50%, used to calculate the sample size of the trial, resulting in a much lower number of samples tested. In fact, during the trial period, the capacity of the RT-LAMP testing station was used less than 50%, making the laboratory cost per test 2,61 EURO higher than it was estimated for the 100% utilization of the testing capacity. We investigated this source of uncertainty in the PSAs by varying the response rate and the implementation costs jointly and our base-case results remained unchanged. In addition, when implemented in a routine setting or in other geographical locations, the response rate could be higher or lower than the response rate we observed during the trial and thus biased the estimates in both directions. We assessed this uncertainty thoroughly in the sensitivity analysis. Last, given that our study adopted a health system perspective, we did not include the costs to the participants. However, this cost is very limited considering the low-barrier to self-sampling, followed by an RT-LAMP analysis and the relatively simple task of sending back the samples by pre-addressed and pre-paid posts.

## Conclusions

The strategy A2 (direct testing of households irrespective of COVID-19 symptoms) appeared to be the most cost-effective of the four active surveillance strategies tested in the Cov-Surv-Study trial. Our findings support the adoption of strategy A2 for routine implementation to strengthen the existing passive surveillance system and facilitate evidence-based decision making by producing a more accurate estimation of the true burden of COVID-19. Future studies should assess the affordability and budget impact of adopting strategy A2 under routine conditions in Germany and similar high-income settings.

### Supplementary Information

Below is the link to the electronic supplementary material.

Supplementary file1 (DOCX 547 KB)

Supplementary file2 (DOCX 174 KB)

Supplementary file3 (DOCX 19 KB)

## Data Availability

Anonymized data on individual level information will be made available upon official request to the University Medicine’s Network (Netzwerk Universitätsmedizin, NUM) for COVID-19 research in Germany. All other data related to this publication will be made available upon a written request to the corresponding author.
