# Supplementary material for: Cost and cost-effectiveness of four different SARS-CoV-2 active surveillance strategies: evidence from a randomised control trial in Germany
Source: Eur J Health Econ. 2023 Jan 19;24(9):1545–59. doi: 10.1007/s10198-022-01561-8 (PMC9850332; doi:10.1007/s10198-022-01561-8)
Supplement: Supplementary file 1 — Supplementary file1 (DOCX 547 KB) [file 10198_2022_1561_MOESM1_ESM.docx]

**Annex 1:** Pre-screening questionnaire for adults used in the Cov-Surv-Study trial in 2020


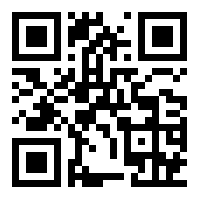
**Symptom Questionnaire**

Webseite:

**(if possible please fill out online at www.virus-finder.de**

**access code: "AccessCode_A")**

**Instructions:** Please fill in with pen. If you ticked something wrong
please fill in this box completely and tick the other one if necessary.

1) Do you have a current flu vaccination (please tick)? Yes No

If yes

If yes

If yes

If yes

If yes

If yes

If yes

If yes

If yes

If yes

If yes

If yes

If yes

If yes

If yes

If yes

I often have this
symptom

2) Have you had one or more of the following symptoms (please tick)?

Temperature/Fever

Cough (dry)

Do you cough up phlegm/mucus?

Sore throat

Shortness of breath

Muscle pain

Tiredness/Fatigue

Headache

Runny nose / cold

Chest pain

Diarrhea

Nausea

Loss of taste or smell

Shivering/Chills

Breathlessness

Disturbance of consciousness

Others: if yes, please specify:

_______________________________________________________________________________


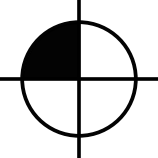
_______________________________________________________________________________

3) Do you practice a medical/nursing profession? YES NO

4) Do you have contact with children/young people in your job? YES NO

5) What is your employment?

__________________________________________________________

6) How many people live in your household?

1 (me) 2 3 4 5 more than 5 (please specify)|__|__|

**The following questions are optional, we would appreciate it if you could answer them anyway:**7) Do you have one or more of the following lung diseases?

Mild bronchial asthma (without regular medication)

Other lung diseases, like

- Asthma with regular medication,
- Chronic obstructive pulmonary disease,
- Interstitial lung disease, lung cancer

8) Do you have high blood pressure? YES NO

9) Have you had a cardiovascular disease (heart attack, stroke etc.)? YES NEIN

10) Do you have a chronic kidney disease? YES NO

11) Do you have diabetes? YES NO

12) Have you had cancer therapy or cancer in the past two years? YES NO

13) What is your highest education level?

PhD

Master studies, diploma studies at a university or college

Bachelor studies at a university or college

Master craftsman training

High school, technical/economic high school, comprehensive school, school with college entrance qualification

Vocational school (dual system), civil servant trainee* in service, one-year training in health/social professions, basic training year

Secondary school, vocational school (middle school leaving certificate), special school

No degree / still in school education

I decline the use of my information for future research purposes.


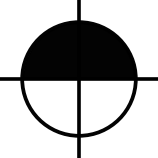
**Please sign the consent form for the voluntary questionnaire below!**

**Consent form**

**Study on novel corona testing strategies for the general population**

The contents of this information brochure were sent to me and can also be viewed on the study's website. I hereby confirm that I have read the information document, or it has been read or explained to me in a suitable language. I have understood the nature, conduct, benefits, and risks of the study as well as the requirements. If I have any questions in this regard, I may contact the telephone hotline mentioned in the information brochure. I have had sufficient time for my decision. I voluntarily agree to participate in the survey**.**

**Data protection**

I am aware that personal data will be processed in this study. The processing of the data is carried out per legal regulations and, according to Art 6. §1 of the German data protection law (germ. DSGVO)”. a, requires a declaration of consent.

I have been informed and voluntarily agree that the data collected in the study as well as the determination of coronavirus (SARS-CoV-2) in my sample, will be recorded and evaluated in a pseudonymous form (i.e. without name, address, telephone numbers, email addresses). This information will be treated confidentially and will be used to contact me if I have been tested positive for coronavirus. Third parties are not allowed to view personal data. When the results of the study are published, only anonymized data will be published. I am aware that this consent may be revoked at any time in writing or verbally without explanation, without any disadvantages to myself. This does not affect the legality of the data processing that took place until the revocation. In this case, I can decide whether the data collected should be deleted or whether it may continue to be used for the study.

**Informing Person**

The information is provided via the information brochure and the website. If the participant has further questions, further information can be found on the website: [www.virus-finder.de](http://www.virus-finder.de), he or she may also contact the study’s organizers via E-mail at: info@virus-finder.de.

**I hereby give my consent to participate in the survey and to process the data within the scope of this study.**

**Date, Signature:_____________________________________**

I decline the use of my information for future research purposes.
