# Supplementary material for: Cost and cost-effectiveness of four different SARS-CoV-2 active surveillance strategies: evidence from a randomised control trial in Germany
Source: Eur J Health Econ. 2023 Jan 19;24(9):1545–59. doi: 10.1007/s10198-022-01561-8 (PMC9850332; doi:10.1007/s10198-022-01561-8)
Supplement: Supplementary file 2 — Supplementary file2 (DOCX 174 KB) [file 10198_2022_1561_MOESM2_ESM.docx]

**Annex 2: Additional detail on the methods**

**Table 1:** Estimates of model parameters, the corresponding mathematical distributions for probabilistic sensitivity analyses and their data sources

| **Model parameters** | **Most likely value and range values by study arms** | | **Mathematical distributions** | **Sources** |
| --- | --- | --- | --- | --- |
|  | **A1** | **A2** |  |  |
| Start-up cost (€) | 40.290 (±20%) | 40.290 (±20%) | Gamma | own costing |
| Implementation costs (€) | 88.343 (±20%) | 73.328 (±20%) | Gamma | own costing |
| Response rate (%) | 41,2 | 36,2 | Beta | Cov-Surv-Study trial |
| Implementation period (month) | 1 - 60 | 1 - 60 | Uniform | Assumption |
| Prevalence (%) | 0,31 (0,01-4) | 0,35 (0,01-4) | Beta | Cov-Surv-Study trial |

**Figure 1:** Structure of the decision tree assessing the cost-effectiveness of the two active surveillance strategies (A1 and A2) in the Cov-Surv-Study trial and the status quo of having no active surveillance for cost per sample tested

**
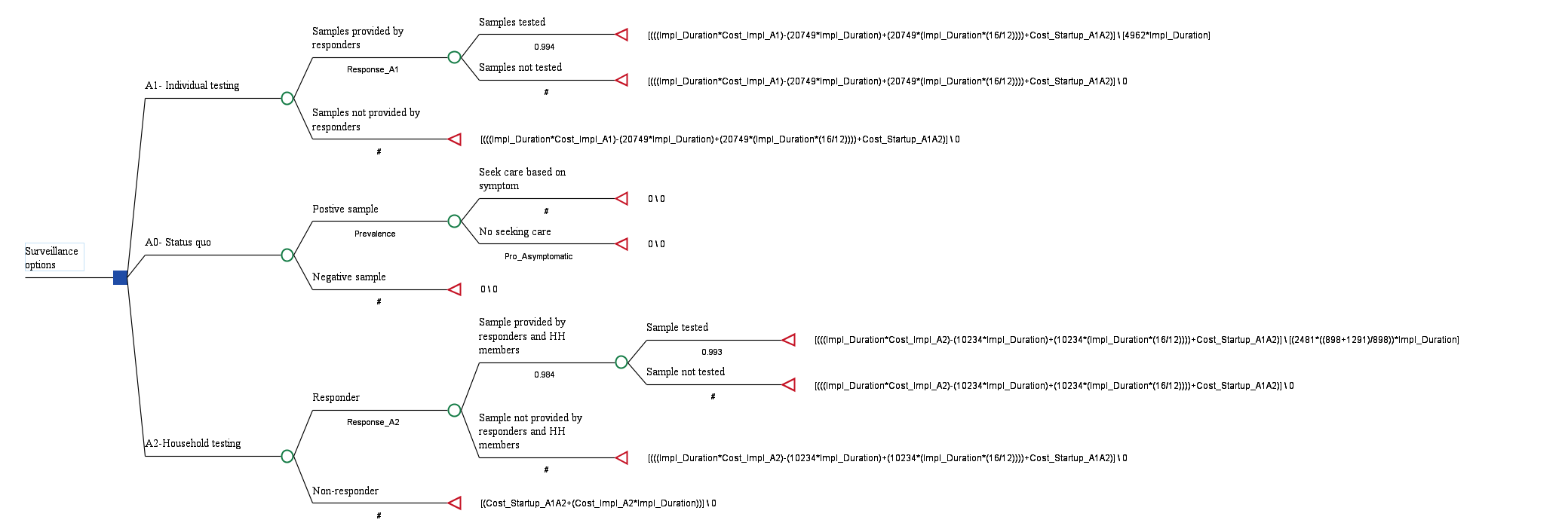
**

**Figure 2:** Structure of the decision tree assessing the cost-effectiveness of the two active surveillance strategies (A1 and A2) in the Cov-Surv-Study trial and the status quo of having no active surveillance for cost per case detected

**
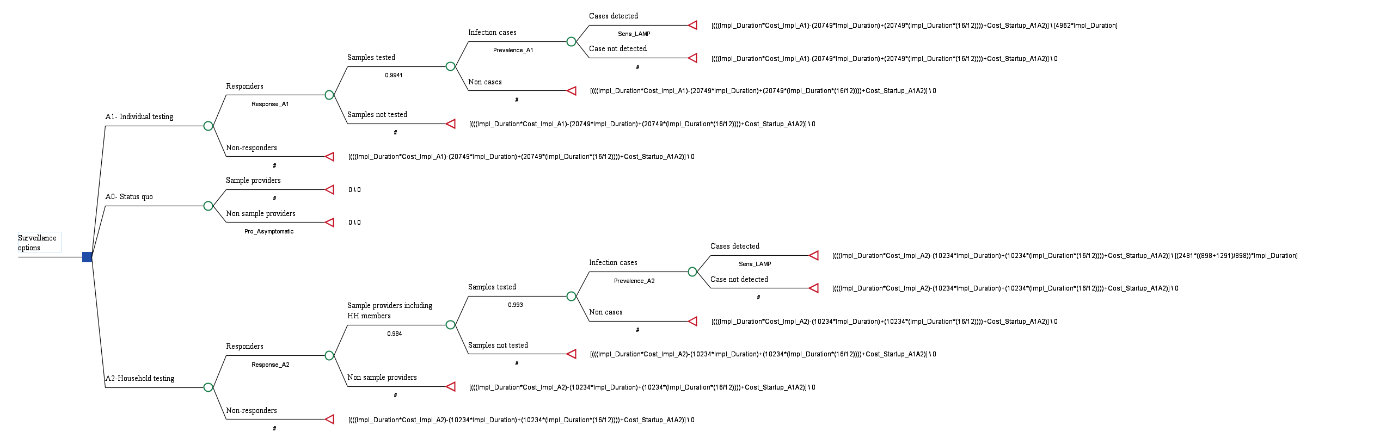
**
