# Supplementary material for: Cost and cost-effectiveness of four different SARS-CoV-2 active surveillance strategies: evidence from a randomised control trial in Germany
Source: Eur J Health Econ. 2023 Jan 19;24(9):1545–59. doi: 10.1007/s10198-022-01561-8 (PMC9850332; doi:10.1007/s10198-022-01561-8)
Supplement: Supplementary file 3 — Supplementary file3 (DOCX 19 KB) [file 10198_2022_1561_MOESM3_ESM.docx]

**Annex 3: Additional detail on the results**

**Table 1: Cost-effectiveness results of the four SARS-CoV-2 active surveillance strategies estimated for the implementation period extended to 60 months**

| **Cost-effectiveness results** | **Surveillance strategies** | | | |
| --- | --- | --- | --- | --- |
|  | **A1** | **A2** | **B1** | **B2** |
| **Cost estimates in EURO** |  |  |  |  |
| Start-up costs | 40.290 | 40.290 | 52.077 | 52.077 |
| Implementation costs | **5.715.581** | **4.604.349** | **5.647.105** | **3.686.270** |
| Total costs | **5.755.871** | **4.644.639** | **5.699.182** | **3.738.347** |
| **Outcome estimates** |  |  |  |  |
| Number of responders recruited | 122.580 | 53.880 | 295.560 | 140.400 |
| Number of samples tested*** | 121.860 | 128.340 | 33.300 | 35.640 |
| Number of cases detected | 360 | 420 | 180 | 60 |
| Number of cases could have been detected* | 60 | 0 | 180 | 180 |
| Number of asymptomatic cases | 180 | 180 | n/a | n/a |
| **Average cost per outcome in EURO** |  |  |  |  |
| Cost per responder recruited | 46,96 | 86,20 | 19,28 | 26,63 |
| Cost per sample tested | 47,23 | 36,19 | 171,15 | 104,89 |
| Cost per case detected | **15.989** | **11.059** | **31.662** | **62.306** |
| Cost per case detected all** | **13.704** | **11.059** | **15.831** | **15.576** |
| Cost per asymptomatic case detected | 31.977 | 25.804 | n/a | n/a |

* *Cases which reported to test positive two weeks before the trial and therefore declined to participate*

*** Including cases which have declined to participate because of having positive test two weeks before the trial*

*^***^ Consisting of samples provided by responders (A1 and B1) and their household members (A2 and B2)*

**Table 2**: **Cost-effectiveness results of the four SARS-CoV-2 surveillance strategies estimated for the response rate decreased to 20%**

| **Effectiveness results** | **Surveillance strategies** | | | |
| --- | --- | --- | --- | --- |
|  | **A1** | **A2** | **B1** | **B2** |
| Number of responders recruited | 1.610 | 753 | 4.133 | 2.003 |
| Number of samples tested*** | 1.600 | 1.792 | 466 | 508 |
| Number of cases detected | 4,73 | 5,87 | 2,52 | 0,86 |
| Number of cases could have been detected* | 0,79 | 0,00 | 2,52 | 2,57 |
| Number of asymptomatic cases detected | 2,36 | 2,51 | n/a | n/a |
| **Average cost per outcome** |  |  |  |  |
| Cost per responder | 78,49 | 60,26 | 32,61 | 46,47 |
| Cost per sample tested | 78,95 | 61,72 | 289,39 | 210,17 |
| Cost per case detected | 26.724 | 18.940 | 53.538 | 125.473 |
| Cost per case detected all** | 22.907 | 18.940 | 26.769 | 31.368 |
| Cost per asymptomatic case detected | 53.449 | 44.194 | n/a | n/a |

* *Cases which reported to test positive two weeks before the trial and therefore declined to participate*

*** Including cases which have declined to participate because of having positive test two weeks before the trial*

*^***^ Consisting of samples provided by responders (A1 and B1) and their household members (A2 and B2)*
